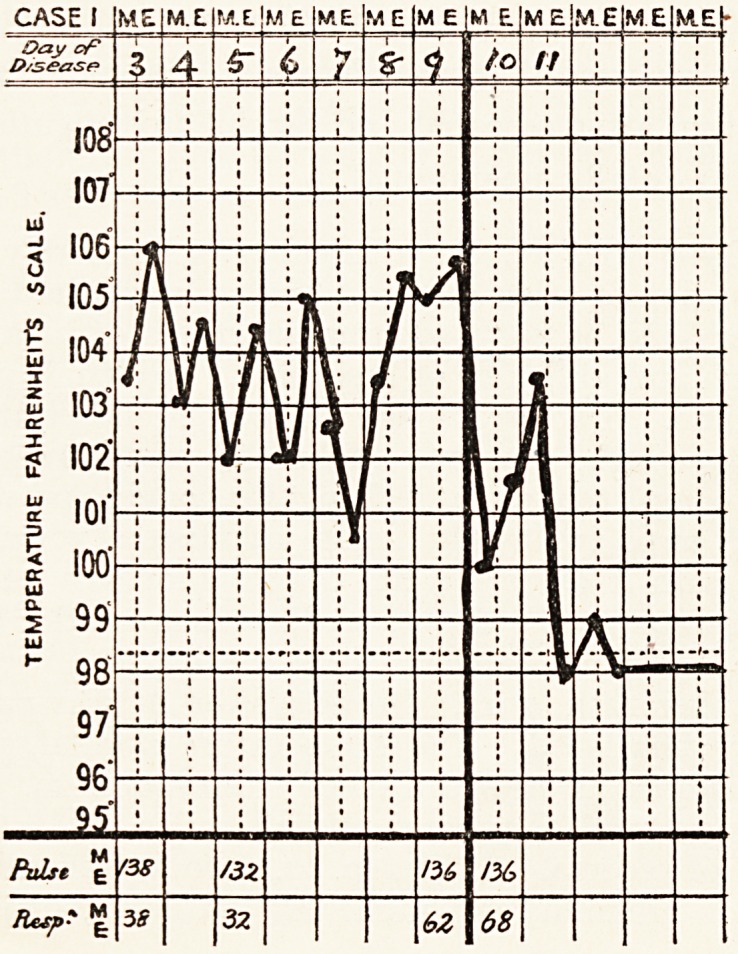# Diseases of the Lungs Associated with the Presence of Friedländer's Bacillus

**Published:** 1914-03

**Authors:** J. Michell Clarke

**Affiliations:** Professor of Medicine, University of Bristol; Physician to the Bristol General Hospital


The Bristol

Medico-Chirurgical Journal.

" Scire est nescire, nisi id me
Scire alius sciret."

MARCH, 1914.

### DISEASES OF THE LUNGS ASSOCIATED WITH THE PRESENCE OF FRIEDLANDER'S BACILLUS.

BY

J. Michell Clarke, M.A., M.D., LL.D., F.R.C.P.,

*Professor of Medicine, University of Bristol; Physician to the Bristol General Hospital.*

Several cases of pulmonary disease have recently been under my care in which Friedländer's bacillus was either obtained from the sputum in pure culture, or if in association with other organisms, appeared to be the chief morbific agent. The first question that arises is whether examination of the sputum can give an accurate representation of the process going on in the lung. Although there is considerable difference of opinion on this point, good authorities now hold that if the sputum is examined with certain precautions it does give such a representation. Bulloch[1] states that "in the careful and extended observations of Spengler there appeared to be a close connection between the conditions found in the sputum and lung respectively." In the

[1] *System of Medicine,* Allbutt and Rolleston, vol. v., p. 378.

following cases where there was a mixed infection that organism which was found ingested by the leucocytes was taken to be the important one.

B. Friedländer only accounts for a very small percentage of cases of pneumonia, and in these cases the lobar distribution is often due to an aggregation of lobular patches, with secondary patches of lobular pneumonia around the main area. The alveolar contents are viscid, due to a mucous secretion, the latter mechanically hinders the entrance of leucocytes into the alveoli, and therefore cells and fibrin are scanty.[1] The infiltration of the lung is apt to persist, and solution (lysis) of bacteria to be hindered because the bacilli are very resistant and cause only slight local reaction. Although it seems to be often a very severe affection, yet fever is often lower than in pneumococcal pneumonia, because on account of the slighter local reaction only few bacterial products are dissolved and absorbed. Abel and Hallwachs also state that there is a pronounced tendency to the formation of central patches of softening which may lead to the separation of necrosed patches of lung.

In mixed cultures B. Friedländer overcomes the pneumococcus.

It is well known that B. Friedländer is frequently present in affections of the nose and throat and elsewhere in the body, but in this paper I shall keep to pulmonary conditions. Friedländer's bacillus is found in pulmonary tuberculosis and chronic lung affections, in addition to being an occasional cause of pneumonia.

**Case 1.**—My first case is one of pneumonia of some severity, but of somewhat irregular course, in a girl aged 18, who had had no previous illness except enteric fever five years before. The illness began acutely three days before admission to hospital with vomiting, pains in the

[1] Abel and Hallwachs, *Handb. d. Pathogen. Mikro-organisms*, Jena, 1913, pp. 532-3.

right shoulder, a rigor, and high fever. She had eaten freely of ice cream the evening before the onset. On admission she was flushed, there was an outbreak of labial herpes, some diarrhœa, tongue thickly covered with a creamy white fur, pulse rate 140, respiration 38. Temperature 104°, rising to 106° the same evening.

Examination two days later showed dulness at the apex of the right lung, with bronchial breathing, coarse crepitations, and bronchophony ; there were deficient resonance and air entry at the right base, with coarse moist crepitations. A few scattered finer râles were heard over the base of the left lung. For the first two days there was also some general bronchitis. The heart and abdominal organs were normal ; the urine contained a trace of albumin. She coughed up a quantity of yellowish or yellowish green expectoration. Cultures made from this showed the presence of B. Friedländer and M. catarrhalis.

The illness ended by crisis on the eleventh day. The lung condition apparently cleared up completely, and she made a good recovery.

Differences from pneumococcal pneumonia are seen in the irregular oscillations of the temperature, the characters of the sputum, which was yellowish green, abundant and not blood-stained, and the absence, as shown by physical signs, of dense consolidation. Indeed, the signs at the right apex for two or three days suggested the possible formation of a cavity, and though the lung subsequently appeared to clear up, the case suggests, though it would not be safe to say more, that after such an illness the affected right apex might easily have contained a patch of necrosis, and become the starting-point of future disease, such as was found in case 3 below.

In the next two cases the pulmonary lesions were those typical of chronic pulmonary tuberculosis with excavation. So typical were the lesions in character, and especially in distribution, that from the physical signs the diagnosis of chronic phthisis was confidently made, and when the pathologist reported that no tubercle bacilli could be found in the sputum after repeated and careful investigation, such a result was totally unexpected.

**Case 2.**—J. H., æt. 45, a labourer. No family history of consumption or other disease. He had never had syphilis. Three years ago he suffered from shortness of breath, with some cough and difficulty of breathing. No renal symptoms at that time. Two years ago he was in hospital for six weeks with cough, shor ness of breath, and cramps in the legs. He completely recovered, lost his cough, and remained fairly well until two weeks before admission, when he was taken ill with a shivering fit, cough, shortness of breath, painful cramps in the limbs, and swelling of the legs and feet. He had had diarrhœa for some days.

September 12th, 1912. On admission he was pale, thin, aud badly nourished. The temperature normal, pulse 84, of high tension, arteries thickened, respiration 20. Tongue foul, thickly furred. Legs and ankles swollen, and a "lumbar cushion" of œdema. Urine, sp. gr. 1010, contained a large amount of albumin, and numerous epithelial, hyaline and granular casts. Blood examination: red cells 4,700,000, whites 19,200 to c.m., hæmoglobin 86 per cent. Lungs: right upper lobe normal. At apex of left lung, front and back, there were dulness, bronchial breath-sounds, bronchophony, and some crepitations. At anterior part of axillary region was an area over which was dulness, cavernous breathing, bronchophony and pectoriloquy. At the lower angle of left scapula, over an area about three inches in diameter, were distinct signs of a cavity, *i.e.* high-pitched percussion note, cavernous breathing, liquid râles, post-tussive suction, and bronchophony. Coarse moist râles were heard over the bases of both lungs in front, and over the back of the chest as high as the lower angle of scapula. The heart was slightly enlarged, the blood pressure raised, and the edge of the liver felt about two inches below the ribs.

From the physical signs the condition of the lungs was looked upon as due to chronic tuberculosis with excavation. The sputum was large in quantity, purulent, and slightly offensive. The first culture showed presence of B. Friedländer and M. catarrhalis. Antiseptic inhalations were started from an oronasal inhaler, and carried out almost continuously. A second culture a week later gave a pure growth of B. Friedländer, and this continued to be the case whilst the patient was in hospital. Repeated examinations for T. B. were made, but always with a negative result. There were no elastic fibres in the sputum.

On October 1st a course of injections of a vaccine made from cultures of B. Friedländer was begun. At first five millions were injected, and the dose gradually increased up to five hundred millions. There was a slight local reaction at the site of injection after the first injection, but no reaction of any kind until the last dose was reached. One hour later the patient vomited, shivered, and the temperature rose to 101.8°. The result of treatment by rest, a full dietary, antiseptic inhalations, and specific vaccine was a gain in weight of $1\frac{1}{2}$ stone, reduction of the large amount of purulent sputum to a negligible quantity, and no apparent effect on the kidneys except a slight fall in the amount of albumin in the urine; in physical signs a striking diminution in the moist râles which on admission were so general over the lower part of both lungs, and with this relief of cough and shortness of breath; no effect on the local lesions or cavities, which seemed to be steadily extending and involving fresh portions of lung at their margins. At left apex there were now "box-note" on percussion in front with liquid râles, bronchial breathing, and pectoriloquy. The physical signs at anterior part of left axilla and at lower angle of scapula remained much the same, but audible over a somewhat larger area. It is not possible to say what share in improvement is to be attributed to the vaccines. Although the man had gained weight and improved in some respects, his condition was far from satisfactory when he left the hospital, as the destructive process in the lung was still continuing. Friedländer's bacillus was still present when the sputum was last examined.

**Case 3.**—A. B., æt. 31, cashier. Family history good. He had had two attacks of pneumonia, at the ages of 13 and 19. In the latter attack he was under my care, and on referring to the notes of his case I find that the pneumonia affected the whole of the right lung, and was most pronounced in the upper lobe; that it was of somewhat irregular type, the temperature showing considerable oscillations, and coming down about the sixth day. The lung is stated to have cleared up satisfactorily. He had been in good health since then except for a cough at times. He attributed his present illness to chill. He was ill in bed for a fortnight before admission to hospital with severe pains in right side of chest, cough, large amount of expectoration, shortness of breath, heavy night-sweats, and for the first week his temperature ranged between 101° and 103°.

On admission his temperature was 101.5°, but fell to 99° the next day, and remained between 98° and 99° during the rest of the illness. Pulse 96, respiration 24. Tongue, thick yellow fur, urine normal. He looked thin and ill, there was no cyanosis. The chest was flat, narrow, and showed well-marked lateral groves.

Lungs: dulness over right apex, above clavicle and over first and second intercostal spaces in front and over suprascapular region behind, with bronchial breath-sounds, scattered moist râles, and bronchophony, and at outer anterior part of apex breath-sounds were cavernous, râles metallic, with post-tussive suction and pectoriloquy. On left side, at inner end of second intercostal space, in fifth and sixth spaces in axillary region, were patches of deficient resonance, with small moist crepitation. The breath-sounds were harsh, attended with rhonchi and a few coarse râles over the back of the right lung, as high as lower angle of scapula and over the extreme left base. The heart was normal. The sputum was copious, not offensive, in nummular masses. Repeated examinations for T. B. were negative. Cultures gave the presence of B. Friedländer. He stayed three weeks in the hospital, and made a steady and uneventful progress towards recovery under ordinary treatment. At the end of this time his cough was very slight, and there was practically no expectoration. He gained 5 lb. in weight during the last week of his stay, and felt quite well. The rhonchi and moist sounds had completely cleared up over both lungs, but there remained in the outer part of the right apex, towards the axilla, distinct signs of excavation in the presence of dulness, cavernous breath-sounds, post-tussive suction, and pectoriloquy.

The distribution and character of the lesions in these cases were thus exactly those of progressive pulmonary tuberculosis. This distribution of lesions, originating near the apex, and spreading downwards therefrom, with an early focus of disease in the apex of the lower lobe, is one of the most valuable and distinctive signs in the diagnosis of pulmonary tuberculosis. Further, this distribution of the lesions does not correspond to the ordinary position of bronchiectases, which usually most affect the lower lobes. In the first case, the history of indefinite chest illness well consorts with pulmonary tuberculosis, and the question arises as to the share played by B. Friedländer in the lung destruction. The man's resistance would have been lowered by the existing chronic Bright's disease, and we might suppose that the pulmonary lesions were indeed produced by the B. tuberculosis, and that subsequently the cavities were invaded by the B. Friedländer, which turned out the tubercle bacillus. If this supposition is correct, the patient's condition was no whit the better, for the work of destruction appeared to be actively going on. Such an explanation is more difficult in the second case, for the man was in good health until the onset of the present illness. In this case, however, it is difficult to suppose that a cavity apparently of some size could be produced in a fortnight. The previous history of two attacks of pneumonia is interesting, and also the known fact that the lung completely cleared up after the second attack. Bearing in mind the tendency of the B. Friedländer to cause local softening and necrosis, it is possible that the disease might have been insidiously going on for some time, until exposure to cold induced a sudden and considerable extension of mischief, and that the B. Friedländer might have been the only agent at work. If so, the close resemblance in physical signs to the lesions produced by B. tuberculosis is remarkable. On the whole, I incline to the view that in both cases the older lesions in the apices were produced by B. tuberculosis, and that a secondary infection by B. Friedländer occurred later, and caused the more general and patchy consolidation of the lungs. In both cases the onset of the present illness was marked by a very distinct sudden onset, with chills, fever, pains in the chest, and cough, with a very abundant purulent expectoration. In the subsequent course of the illness in each case, a notable fact is the maintenance of the temperature at or about normal, rarely exceeding 99°, in the presence of extensive excavation of the lungs, extensive areas of broncho-pneumonia, and the expectoration of large quantities of semi-purulent sputum. In the first case, the presence of chronic nephritis with its known tendency to cause a subnormal temperature, might partly account for the absence of hectic fever ; but in the second case there was no such source of explanation. The reason for the apyrexial course of the disease may be found in the general account of some of the charactersitics of B. Friedländer given above.

In the other cases, B. Friedländer was found in association with other micro-organisms, and presumably had engrafted itself on pre-existing chronic pulmonary lesions. Thus in three cases there was fibrosis of the lung, in two accompanied also with bronchiectasis, the sequel of previous attacks of pneumonia. Of these cases in one B. Friedländer was obtained in pure culture from the sputum, in another it was associated with a streptococcus, and in the third with M. catarrhalis. In all the sputum was large in quantity, thick, of a greenish yellow colour, and had a slight fœtor. In two of the cases a vaccine was used.

One patient was a single woman, aged 56. She had had nasal polypi removed, and for many years suffered from dyspepsia, but no other illness, and enjoyed fair health until four years ago, when she had left-sided pneumonia. Since then she had been in poor health, with loss of flesh, cough and expectoration daily of a quantity of muco-pus, which often had a disagreeable smell. She suffered from paroxysmal attacks of dyspnœa, in which she became extremely cyanosed. All symptoms had become aggravated since an attack of influenza six months previously. Examination of the chest showed on the left side thickening of pleura with fibrosis of the lung, and signs of bronchiectasis towards the base at the back, probably originating in an unresolved pneumonia. The heart's apex was displaced outwards from the contraction of the left chest. The other organs and the urine was normal. There was no œdema.

Examination of the sputum showed the presence of B. Friedländer and the pneumococcus. The lung was punctured, and from the material withdrawn the same organisms were cultivated. She was treated by rest, good feeding, intra-tracheal injections of izal and glycerine, and by a vaccine prepared from cultures of B. Friedländer, as Mr. Scott-Williamson considered this to be the active organism. Injections, beginning with a dose of two millions, and increasing up to 500 millions, were given on July 9th, 10th, 11th, 12th, 13th, 20th, 23rd, 24th, 25th, 26th and 27th. The only direct effect noticed was that on two or three occasions there was a little redness and soreness of the arm at the site of injection. There was no change in the condition of the lung. A second mixed vaccine, of B. Friedländer and pneumococcus was then prepared, and five doses, ranging from five to 100 millions were given between August 1st and 5th; again without appreciable effect. She left the hospital on August 8th. The net result of six weeks' treatment was a considerable improvement in general health, a gain of 7 lb. in weight, great improvement in shortness of breath, and disappearance of the distressing attacks of dyspnœa, reduction of the large quantity of sputum to an inappreciable amount, and loss of fœtor of sputum and breath. The physical signs in the lungs remained practically unaltered.

Another of the cases was under the care of Dr. Symes, who has very kindly allowed me to use his notes.

The patient, a woman aged 24, was in hospital in August, 1912, with eclampsia, which was followed by double pneumonia. She was ill eight weeks. Since that time she had constantly coughed up large quantities of frothy greenish yellow sputum with an offensive odour. Repeated examinations for B. tuberculosis had given a negative result. Previous to this illness she enjoyed perfect health. Examination of chest showed fibrosis of the lower lobe of the left lung with bronchiectases. Examination of the sputum showed presence of B. Friedländer and of M. catarrhalis. She was four weeks in the hospital; during the whole time the temperature was normal. A vaccine was prepared from cultures of B. Friedländer, and injected on July 14th, 16th, 18th, 21st, 23rd and 25th, beginning with a dose of five millions and increasing up to two hundred millions. No appreciable effect was observed on the temperature or otherwise from the injections. She gained 5 lb. in weight and improved in general health. The cough was much relieved, the quantity of sputum greatly reduced, and it became much less offensive. No note was made as to the condition of the lung, which was presumably unaltered as to the fibrosis and bronchiectases.

The absence of any fever in both these cases is worthy of note.

In another case a man, whilst eating some chicken soup, swallowed one of the chicken's neck vertebræ ; he felt it go "the wrong way;" and had a violent fit of choking and coughing, with subsequent pain in the upper part of the left chest. I saw him about eight months afterwards. He was then emaciated, weak, had a severe paroxysmal cough, and coughed up 8 to 12 oz. daily of yellowish offensive sputum. Examination showed deficient expansion, air entry, and resonance over the left lung, front and back, except the upper part of the upper lobe, over which the breath sounds were bronchial. Rhonchi were heard over the whole lung, with a few moist sounds at the extreme base. At the lower part of the left interscapular region, over an area of three inches in diameter, there was dulness, absent breath sounds at times, at others distant bronchial breathing, and increased vocal resonance. There was no fever, just as in the other cases. Friedländer's bacillus, pneumococci, and M. catarrhalis were found in the sputum. Mr. Scott-Williamson considered the B. Friedländer to be the important organism, and although I was not very sanguine as to any good results whilst the bone was still there, I thought that a vaccine might possibly improve the condition of the surrounding lung. A course of vaccines was therefore given, but without result. The subsequent history is interesting. His wife came to visit him whilst she had acute influenza ; he contracted it from her, and the course of the illness entirely changed. For the next four weeks he had a swinging temperature of 100° a.m., to 102° or 103° p.m. Loud bronchial breathing and moist coarse crepitations appeared over the area of consolidation in the interscapular space. Examination of the sputum gave a pure culture of the B. influenzæ, which thus appeared to have turned out the other organisms. He insisted on going home, got gradually better, and one Sunday whilst in church he had a violent paroxysm of cough, and coughed up the chicken's vertebræ, which he brought up to show me.

I gratefully express my thanks to Mr. Scott-Williamson for his careful bacteriological examinations in these cases.

## Figures and Tables

**Figure f1:**
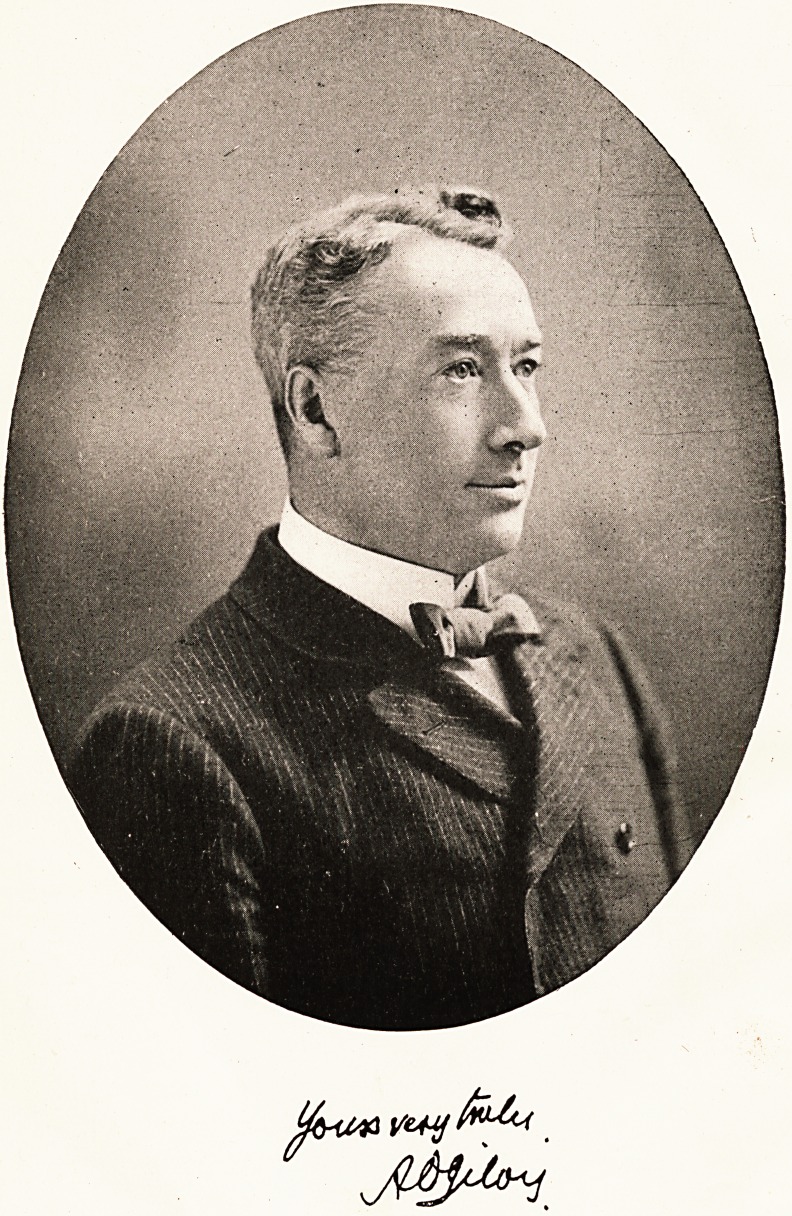


**Figure f2:**